# Rapid and accurate genotyping of human SNP rs671 in aldehyde dehydrogenase 2 gene using one-step CRISPR/Cas12b assay without DNA amplification

**DOI:** 10.1186/s13008-023-00095-6

**Published:** 2023-08-28

**Authors:** Fang Wu, Yong Xue, Yan Wang, Xinxin Si, Xinyue Zhang, Yuyang Xu, Zhidan Luo

**Affiliations:** 1 Jiangsu Key Laboratory of Marine Biological Resources and Environment, Jiangsu Key Laboratory of Marine Pharmaceutical Compound Screening, Jiangsu Ocean University, Lianyungang, 222005 China; 2 Co-Innovation Center of Jiangsu Marine Bio-Industry Technology, Jiangsu Ocean University, Lianyungang, 222005 China; 3 Department of Pathology, The Second People’s Hospital of Lianyungang, Lianyungang, 222001 China

## Abstract

**Background:**

The SNP rs671 of Human aldehyde dehydrogenase (ALDH) is G-A transition at 1510th nucleotides, which is an important clinical indicator of alcoholic liver disease, digestive tract cancer and some drug efficiency. The commonly used genotyping assay of this polymorphism is relatively time-consuming and costly.

**Finding:**

This study develops a rapid and accurate one-step CRISPR/Cas12b assay to distinguish the G1510A polymorphism of human ALDH2 free of DNA amplification. The method we established requires only one step of adding 1 μl genomic DNA sample to premixed system, and waiting for the acquisition of fluorescent signal, taking approximate 30 min.

**Conclusions:**

This method provides a potential tool for more accurate and reliable nucleic acid detection with a single base difference and supports the relevant disease diagnosis and personalized medicine.

**Supplementary Information:**

The online version contains supplementary material available at 10.1186/s13008-023-00095-6.

The commonly clinical used genotyping assay of single nucleotide polymorphism (SNP) is relatively time-consuming and costly. We developed a rapid and accurate one-step CRISPR/Cas12b assay to distinguish homozygote and heterozygote of SNP rs671 of human aldehyde dehydrogenase 2 (ALDH2) gene without any DNA amplification. This assay requires only one step of adding at least 10^3^ copies of genomic DNA sample to premixed system, and taking approximate 30 min to get results. It provided a potential tool for more efficiency and reliable SNP detection method and supported the relevant disease diagnosis and personalized medicine.

Human aldehyde dehydrogenase (ALDH) is a family of enzymes that catalyzes the oxidation of acetaldehyde and other aliphatic aldehydes. Among 19 identified ALDH isoenzymes, the mitochondrial isoenzyme ALDH2, which highly expressed in the liver and stomach, is one of the key enzymes in the human ethanol metabolism pathway [1]. The most clinically significant SNP of ALDH2 gene is rs671 that located in the 12th exon and involved the transition of 1510G to A. This SNP exhibits a minor allele frequency (MAF) of approximately 0.2 in East Asian populations, while being rarely observed in other geographical populations (https://www.ncbi.nlm.nih.gov/snp/rs671). The transition of G to A of rs671 leads to substitution of Glu487 to Lys, that exhibits a serious loss of ALDH2 enzyme activity and is consequently closely related to alcoholic poisoning, alcoholic liver disease, digestive tract cancer, and other diseases [2]. Genotyping of ALDH2 rs671 SNP is also very important in precision medicine to predict the efficacy of nitroglycerin in the treatment of angina pectoris [3].

With the development of molecular biology, a large number of genotyping methods for SNP have been developed. For example, DNA sequencing, the golden-standard assay for genetic diagnosis, is often used but is also time-consuming and costly. Fluorescence probe PCR is the most widely used assay in clinical practice, however, it requires a long detection time and high sample quality [4]. Other existing detection methods for ALDH2 SNP include chain reaction-restriction fragment length polymorphism (PCR–RFLP), denaturing gradient gel electrophoresis (DGGE), denatured high-performance liquid chromatography (DHPLC), and hybridization-based methods, etc. [5–7]. These approaches are not suitable for clinical application due to their inconvenient operation, higher cost, or lack of sufficient accuracy.

Recent advances in the diagnostics field have highlighted the role of clustered regularly interspaced short palindromic repeats-associated (CRISPR/Cas) method as a promising candidate for rapid nucleic acid detection owing to its high reliability and specificity [8]. Several types of Cas nucleases, such as Cas12a, Cas12b, and Cas13a, are employed in these systems. Cas12b is an RNA-guided endonuclease, and could accurately bind to and cleave target double-stranded DNA under the guidance of single-stranded guide RNA (sgRNA). Compared with the previous CRISPR/Cas12a detection method, CRISPR/Cas12b could hardly tolerate any mismatches between sgRNA and target DNA sequences, endowing higher specificity and accuracy when used for SNP detection [9, 10]. The accurate identification of SNP was achieved by Cas12b cis-cleavage and trans-cleavage activities activated by the specific recognition of sgRNA with target DNA. The trans-cleavage activities of Cas12b can cleave non-specific single-stranded DNA (ssDNA) efficiently, such as fluorophore and quench group-modified ssDNA probes. Thus, through cutting the fluorophore and quencher labeling of the ssDNA, the presence of the target gene can be converted into a fluorescent signal.

The principle of the CRISPR/Cas12b-mediated ALDH2 rs671 SNP genotyping system was illustrated in Fig. 1A. The system consisted of an AapCas12b protein (BestEnzymes Biotech Co., Ltd., Lianyungang, China), ALDH2 rs671 SNP-targeting sgRNA, and the FQ reporter probe, which could be premixed in a tube. To differentiate polymorphism of 1510G from 1510A, a 1510G-specific sgRNA was designed. Fortunately, there were no other clinically significant SNP sites within 13 bp each upstream and downstream of rs671 to interfere with the sgRNA design. Sample DNA was mixed with Cas12b proteins, the FQ probe and sgRNA, and subjected to fluorescence signal acquisition. With the presence of human genomic DNA, Cas12b/sgRNA bound to the target sequence forming a ternary complex, which in turn activates the trans-cleavage activity of Cas12b and subsequently cleaves the FQ reporter to produce a fluorescence signal for the detection.

**Fig. 1:**
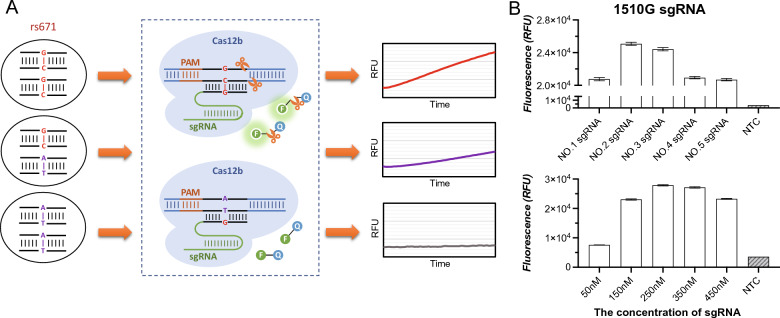
Establishment of the CRISPR/Cas12b assay for genotyping of ADLH2 rs671 SNP. **A** Schematic diagram of the CRISPR/Cas12b method. **B** Optimization of sgRNA conditions, including screening of five candidate sgRNAs and their optimal concentration. Error bars represent show the mean ± standard deviation from three replicates

CRISPR/Cas12b nuclease requires a protospacer-adjacent motif (PAM) sequence TTN for double-strand DNA cleavage, and the PAM sequence should be located in an appropriate position [11]. The distance of the PAM sequence from the start site of the target sequence and the target sequence specificity can also influence recognition efficiency. We analyzed the sequence of ALDH2 gene and found a unique PAM sequence TTG located in 1480–1483 just upstream of rs671 site. Then five sgRNAs were designed and prepared to select the one with the highest cleavage efficiency (Additional file 1: Table S1). Refer to AapCas12b product instruction, the 20 μL reaction mixture contained 600 nM AapCas12b protein, 280 nM sgRNA, 10 × Cas reaction Buffer, 20 U RNase inhibitor (RI), and 100 pg ALDH2 gene fragment containing 1510G were used as template in this study. The reaction mixtures were then quickly transferred to CFX Connect Real-Time System (Bio-RAD, USA) and incubated at 48 ºC. Fluorescence signals were collected every 30 s for a total of 30 min. As shown in Fig. 1B, No.2 sgRNA for 1510G had the highest fluorescence signals and the best detection efficiency. Then the detection system was further optimized with the sgRNA concentration (ranges from 50–450 nM), and 250 nM sgRNA was finalized as the detection reaction condition (Fig. 1B).

The three diploid genotypes of ALDH2 rs671 SNP are homozygous 1510GG, heterozygous 1510GA and homozygous 1510AA. To verify the performance of the method to distinguish the three genotypes, 10^4^ copies of the human genomics DNA containing ALDH2 1510GG, 1510AA, and 1510GA were tested in the assay, respectively. There was a weak signal under 4 × 10^3^ RFU for DNA sample containing homozygous AA, while fluorescence signal dramatically up to more than 20 × 10^3^ RFU for DNA sample containing homozygous GG. For heterozygous GA, the increasing signal falls in between homozygous GG and AA (12 × 10^3^). These results suggested that Cas12b-mediated assay could distinguish the three genotypes of ALDH2 rs641 (Fig. 2A).

**Fig. 2:**
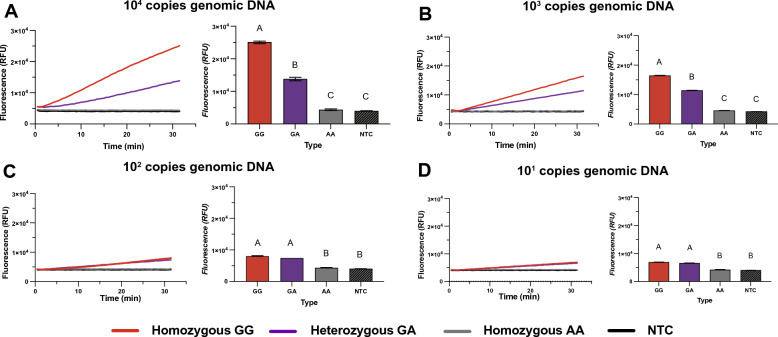
Performance and sensitivity of CRISPR/Cas12b assay for three genotypes of ALDH2 rs671 SNP. The panels represent tenfold gradient concentrations of human gDNA: **A** 10^4^ copies/rxn, **B** 10^3^ copies/rxn, **C** 10^2^ copies/rxn and **D** 10^1^ copies/rxn. For each panel, left is fluorescence curves for 30 min continuous acquisitions, and the right bar graph represents the fluorescence values at the endpoint of 30 min. Error bars represent show the mean ± standard deviation from three replicates. Multiple comparisons between different groups were performed by one-way ANOVA. The groups exhibiting significant differences are annotated with distinct Latin letters, whereas groups with no statistically significant differences are labeled with the same Latin letter

The limit of detection (LOD) of this assay was also determined by tenfold gradient concentrations of human gDNA at 10^3^, 10^2^, and 10 copies per reaction and compared to previous 10^4^ copies/rxn (commonly clinical used concentration). When the genome DNA was 10^3^ copies, the method was also able to distinguish three genotypes of ALDH2 rs671 SNP (Fig. 2B). If the amount of genomic DNA dropped below 10^2^ copies, the fluorescence signal showed no significant difference between homozygous GG and heterozygous GA after 30 min, even the signal of them still higher than homozygous AA (Fig. 2C and D). Therefore, the LOD of this assay was 10^3^ copies/rxn of human genomic DNA, which is sufficient to detect clinical samples.

To analyze the accuracy of our CRISPR/Cas12b-assisted assay, we compared the detection results of clinical blood samples between this method and fluorescence probe qPCR. 10 genomic DNA samples provided by the Second People’s Hospital of Lianyungang (Lianyungang, China) were analyzed and evaluated. The reference fluorescence probe qPCR method was carried out in hospital with Human ALDH2 Gene Polymorphism Detection Kit (Wuhan YZY Medical Science and Technology Co., Ltd., Wuhan, China) that had been extensively used for ALDH2 rs671 SNP genotyping in clinical practice. In a clinical setting, methods of DNA isolation and preparation, quality of reagents, instrumentation may differ and significantly affect fluorescence output, so we add standard plasmid controls (10^4^ copies for each) including the three diploid genotypes of ALDH2 rs671 SNP. The CRISPR/Cas12b-assisted method showed a high level of concordance with the reference fluorescence probe qPCR method (Fig. 3 and Table 1), indicating its potential suitability for the clinical detection.

**Fig. 3:**
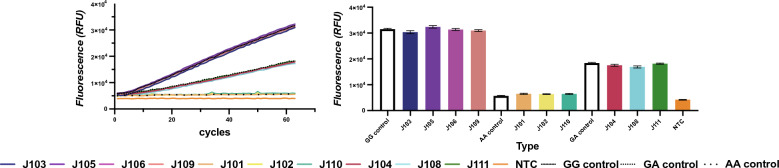
Genotyping of ADLH2 rs671 SNP from twelve clinical blood samples and standard plasmid control using CRISPR/Cas12b assay. Fluorescence curves and the endpoint value of 30 min continuous acquisitions were presented left and right panel, respectively. Error bars represent show the mean ± standard deviation from three replicates

**Table 1:** Genotyping results of ALDH2 rs671 SNP with fluorescence Probe qPCR and CRISPR/Cas12b assays

Sample No	Genotyping results of ALDH2 rs671 SNP	
	Fluorescence probe qPCR	CRISPR/Cas12b
J501	AA	AA
J502	GG	GG
J503	GG	GG
J504	GA	GA
J505	GG	GG
J401	GG	GG
J402	GG	GG
J403	GA	GA
J404	GG	GG
J405	GG	GG
J406	AA	AA
J407	GG	GG

In this study, we established an accurate, rapid, and convenient CRISPR/Cas12b-assisted method to distinguish three genotypes of ALDH2 rs671 SNP. This method only requires one step of adding 30–40 ng (equivalent to 10^4^ copies) of human genomic DNA extracted from clinical sample to a premixed system and waiting for a total of 30 min for the acquisition of fluorescence signal. We didn’t combine any amplification step before Cas12b-mediated genotyping, since copy number of the clinical DNA samples is much higher than the lowest sensitivity of the method. Cas12b could hardly tolerate any mismatch between sgRNA and target DNA sequences, endowing higher specificity and accuracy when used for DNA polymorphism detection. Compared with traditional methods, this method does not require a complicated experimental operation, complex instrument, or long-time course. We believe that the CRISPR/Cas12b genotyping assay will play an irreplaceable role in the gene diagnosis and accurate treatment of clinical diseases.

### Supplementary Information

**Additional file 1: Table S1.** Sequences of sgRNA candidates in this study.

## Data Availability

Not applicable.
