# Supplementary material for: Rapid and accurate genotyping of human SNP rs671 in aldehyde dehydrogenase 2 gene using one-step CRISPR/Cas12b assay without DNA amplification
Source: Cell Div. 2023 Aug 28;18:14. doi: 10.1186/s13008-023-00095-6 (PMC10464061; doi:10.1186/s13008-023-00095-6)
Supplement: Supplementary file 1 — Additional file 1: Table S1. Sequences of sgRNA candidates in this study. [file 13008_2023_95_MOESM1_ESM.docx]

**Table S1 Sequences of sgRNA candidates in this study**

| oligo names | Sequences(5’-3’) |
| --- | --- |
| No.1 sgRNA | GGGGUCUAGAGGACAGAAUUUUUCAACGGGU  GUGCCAAUGGCCACUUUCCAGGUGGCAAAGCC  CGUUGAGCUUCUCAAAUCUGAGAAGUGGCAC  CGGGCUGCAGGCAUACACUG |
| No.2 sgRNA | GGGGUCUAGAGGACAGAAUUUUUCAACGGGU  GUGCCAAUGGCCACUUUCCAGGUGGCAAAGCC  CGUUGAGCUUCUCAAAUCUGAGAAGUGGCAC  GCAUACACUGAAGUGAAAAC |
| No.3 sgRNA | GGGGUCUAGAGGACAGAAUUUUUCAACGGGU  GUGCCAAUGGCCACUUUCCAGGUGGCAAAGCC  CGUUGAGCUUCUCAAAUCUGAGAAGUGGCAC  UGCAGGCAUACACUGAAGUG |
| No.4 sgRNA | GGGGUCUAGAGGACAGAAUUUUUCAACGGGU  GUGCCAAUGGCCACUUUCCAGGUGGCAAAGCC  CGUUGAGCUUCUCAAAUCUGAGAAGUGGCAC  ACACUGAAGUGAAAACUGUG |
| No.5 sgRNA | GGGGUCUAGAGGACAGAAUUUUUCAACGGGU  GUGCCAAUGGCCACUUUCCAGGUGGCAAAGCC  CGUUGAGCUUCUCAAAUCUGAGAAGUGGCAC  UGAAGUGAAAACUGUGAGUG |

**The underlined sequences represent spacer sequences of sgRNA.**
